# Buffalo Medical Association

**Published:** 1879-12

**Authors:** 


### Society Reports.

---

### BUFFALO MEDICAL ASSOCIATION.

*Stated Meeting, November 5, 1879.*

THE PRESIDENT, DR. LUCIEN HOWE, IN THE CHAIR.

Members present, Drs. Moody, Abbott, Trowbridge, Bartlett, White, O'Brian, Brecht, Rochester, Hauenstein, Davidson, Harvey, Little, Hopkins, Wyckoff, Keene, Hartwig, Mynter, Johnson, Fowler, and by invitation, Drs. Granger and Kilbourne, and Mr. J. H. Dormer.

After the transaction of some routine business the Association listened to Professor Rochester's paper on " Pulmonary Diseases of Elevator Employees." (See Original Communications.)

In the discussion which followed, Professor White said: " Medical men are charged with an important duty of pointing out to the community evils which may affect the public health or that of any class of citizens. We should not only inform the public that a new disease has made its appearance among those engaged in a particular business, but should go back further, investigate its causes and suggest such measures as would tend to diminish its frequency or prevent it entirely."

He had long since recognized the existence of such troubles among elevator employees as were described by Dr. Rochester, but now for the first time heard the symptoms described and classified. He was of the opinion that the heated air, made poisonous by the want of a sufficient supply of oxygen, was as important a factor in the production of this form of pulmonary disease as dust.

The men, in large gangs, enter the holds of vessels preparatory to unloading, work hard for many hours in succession, without rest, or little, if any, food. He had long ago suggested that this class of laborers wear vails covering the face, which would, in a great measure, exclude the dust from the lungs, and that an abundance of fresh air be forced in the hold, to replace the foul air and carry off a large amount of the dust. He believed, if the Board of Health could be made to appreciate the alarming fatality among scoopers, it might adopt measures compelling elevator owners to ventilate the holds of their vessels. There was no doubt in his mind that pulmonary disease was produced by such causes, and he was glad that Dr. Rochester had shown to what extent this class of laborers was obliged to endanger their lives.

Dr. Bartlett said during a practice of twenty-five years he had seen a great many of these cases, and urged the men to wear a sponge over the nose and mouth while shoveling, but he believed this had not been fully carried out, it being a source of much discomfort during active labor. He thought Dr. White's suggestion certainly worthy of consideration. These people usually live for the present and have no future; they are dependent on their employers and usually lead dissipated lives. The needle-grinders of Sheffield prefer a short life with good pay to a long one and small wages. These scoopers appear to entertain similar ideas.

Dr. Mynter fully recognized the fact that particular occupations predispose to special diseases. In the example under consideration he looked upon the dust as the exciting cause. Laborers at other kinds of work, in iron foundries for instance, often endure intense heat without permanent injury to health. When shut up in the hold of a vessel, the dust alone would be sufficient to produce serious consequences. The subject seemed to him one of much importance, and well worthy of the attention of the Board of Health.

Dr. Trowbridge's experience would force him to accord with the views of Dr. Rochester. He was quite conversant with the habits of this class of laborers. It so happened that he tallied the first load of corn shipped from Ohio to this port. As a surgeon of the Board of Enrollment, during the last war, he had noticed, more than once, the morbid condition described by

Professor Rochester. Whatever might be the immediate exciting causes of the trouble, he thought that the avarice of the employers, especially the "bosses," and the desire of the men for high wages and whisky, were important factors in the problem. The laborers usually boarded with the "boss;" he induced them to drink as much as they wished, and then worked them to death to get his pay. Until this system is changed, no amount of observation, or research, or philosophizing would have any permanent effect.

Dr. Davidson spoke of the value of the paper as an original contribution to sanitary knowledge. It seemed to him that the irritating particles, composing the dust, must be looked upon as the direct cause. Sponges over the mouth and nose were very good theoretically, but practically they were thrown away as too great an obstruction to the breathing. By the use, however, of such a respirator, as Tyndall has recommended, he thought the workmen might be caused to breathe air more free from the suspended matter.

Dr. Wyckoff was of the opinion that the hours of labor should be shortened, but realizes the difficulty of bringing this about as long as the demands of trade were so imperious.

Mr. Dormer, being called upon by the President, replied that he did not think much could be done to lessen the danger, unless the owners of elevators could be made to appreciate the importance of the matter, and their sympathy and co-operation secured.

He had observed that the hospitals provided for a great many of these people while ill, and the fatality among them was very great. When death or long continued sickness ensued, those dependent upon them for support were thrown upon the mercy of our charitable institutions. The Orphan Asylums and Old Folks' Home were largely filled with them, and the burden of taxation greatly increased in consequence.

Some time ago a committee was appointed by the Charity Organization Society to confer with the owners of elevators to make arrangements whereby these evils might be corrected. A committee was appointed by them to consider the subject, but they have never moved in the matter, and there it rests. In their ambition to rush business they would rather their men would break down than their elevator machinery.

Dr. Rochester, in closing the discussion, said perhaps he did not emphasize as strongly as he might the important effect of other causes besides the dust. The physical strain, long continued, appeared to be an element worthy of consideration. In order that the steam shovelers may not be a moment idle, the men keep at work hour after hour, with hardly a moment's rest, frequently not stopping to eat, but only resting to take a glass of whiskey or a bite of something, and then beginning again. Among the elevators he knew of one, however, where the men were not permitted to work more than twelve hours a day.

Dr. White moved that a committee of five be appointed, of which Dr. Rochester should be chairman, who should take the subject of his paper into consideration, enlist public sympathy in behalf of a reform, and if necessary, strive to secure such legislation as may tend to prolong the lives of elevator employes. Carried.

Dr. Howe subsequently chose Drs. Rochester, White, O'Brien, Davidson and Hauenstein as the gentlemen to constitute the committee.

Under the head of miscellaneous business, a communication was read from J. N. Larned, Superintendent of the Young Men's Association Library, stating that the Library Committee desires to make such purchases of books and periodicals as would be serviceable to medical students and practitioners, the cost of which would not exceed the means placed at their disposal. The opinion of the Buffalo Medical Association was therefore asked, concerning the plan and instructions requested regarding the selection of books.

On motion of Dr. White, the communication was received and the thanks of the Association tendered to the Library Committee, for their generous offer.

Dr. Wyckoff moved that a committee of five be appointed by the President to confer with the Library Committee in the choice of the publications.

Dr. Howe afterwards selected as such committee Drs. Wyckoff, Rochester, Lothrop, Abbott and Bartlett.

In the usual report on prevailing diseases, cases of measles and scarlet fever were mentioned as being particularly frequent, but not of a severe type.

The essayist for the next meeting, on Tuesday, Dec. 2d, was announced by the President as Judge G. W. Clinton, whose subject would be "Malpractice."

On motion, the Association adjourned.

